# Using the MR-Base platform to investigate risk factors and drug targets for thousands of phenotypes

**DOI:** 10.12688/wellcomeopenres.15334.2

**Published:** 2019-11-07

**Authors:** Venexia M Walker, Neil M Davies, Gibran Hemani, Jie Zheng, Philip C Haycock, Tom R Gaunt, George Davey Smith, Richard M Martin

**Affiliations:** 1 Medical Research Council Integrative, Epidemiology Unit, University of Bristol, Oakfield House, Oakfield Grove, Bristol, BS8 2BN, UK; 2 Bristol Medical School: Population Health Sciences, University of Bristol, Oakfield House, Oakfield Grove, Bristol, BS8 2BN, UK; 3 National Institute for Health Research Bristol Biomedical Research Centre, University of Bristol, Oakfield House, Oakfield Grove, Bristol, BS8 2BN, UK

**Keywords:** Mendelian randomization, GWAS, causal inference, causality, sensitivity analysis, genetics

## Abstract

Mendelian randomization (MR) estimates the causal effect of exposures on outcomes by exploiting genetic variation to address confounding and reverse causation. This method has a broad range of applications, including investigating risk factors and appraising potential targets for intervention. MR-Base has become established as a freely accessible, online platform, which combines a database of complete genome-wide association study results with an interface for performing Mendelian randomization and sensitivity analyses. This allows the user to explore millions of potentially causal associations. MR-Base is available as a web application or as an R package. The technical aspects of the tool have previously been documented in the literature. The present article is complementary to this as it focuses on the applied aspects. Specifically, we describe how MR-Base can be used in several ways, including to perform novel causal analyses, replicate results and enable transparency, amongst others. We also present three use cases, which demonstrate important applications of Mendelian randomization and highlight the benefits of using MR-Base for these types of analyses.

## Introduction

Mendelian randomization (MR) is a causal inference method used to study the effects of risk factors and exposures on outcome traits by exploiting genetic variation to address confounding and reverse causation ^1^. Two-sample Mendelian randomization is an extension of this method that allows the use of summary statistics from genome-wide association studies (GWASs) in place of individual-level genetic data. Mendelian randomization can be used across multiple health outcomes for several applications, as detailed in Box 1. However, the data required to perform the analysis and knowledge of the latest methods can be inaccessible. MR-Base ^2^ combines a database of summary statistics on traits and health outcomes from over 20,000 GWASs, with an interface for performing two-sample Mendelian randomization to simplify the implementation of this method. As of February 2019, the repository was populated by curated and harmonized datasets corresponding to over 250 billion single nucleotide polymorphisms (SNP)-trait associations ^3^. Traits include anthropometric measures, risk factors, metabolites, metals, vitamins, circulating proteins, disease outcomes, and disease intermediates (such as LDL cholesterol and systolic blood pressure), as well as DNA methylation and gene expression phenotypes. MR-Base is available via a web interface or through the package ‘ TwoSampleMR’ for R. Useful links, including these, can be found in Box 2.

Box 1. **Applications of MR-Base**

Subject to suitable data and appropriate methods being available, Mendelian randomization can be implemented across multiple health outcomes to:

- Identify novel (or confirm previously reported) risk and prognostic factors
- Evaluate potential interventions for follow-up in independent replication or experimental studies, based on robust causal analysis and data-integration across multiple study designs, without exposure of patients
- Predict unexpected effects (adverse and beneficial) of an intervention
- Provide causal estimates based on exploratory analyses from clinical trials
- Investigate potential biological mechanisms underpinning risk factor-disease associations

Box 2. **Useful links**

- MR-Base web application: http://www.mrbase.org/
- Exemplar code for the use cases: https://github.com/MRCIEU/mrbase_casestudies
- MR-Base PheWAS web application: http://phewas.mrbase.org/
- TwoSampleMR R package: https://github.com/MRCIEU/TwoSampleMR/
- MRInstruments R package: https://github.com/MRCIEU/MRInstruments/
- TwoSampleMR R package wiki: https://mrcieu.github.io/TwoSampleMR/
- Mendelian randomization primer: https://youtu.be/LoTgfGotaQ4
- Mendelian randomization podcast: https://soundcloud.com/bmjpodcasts/mendelian-randomisation-for-the-moderately-intelligent
- Mendelian randomization webinar: https://www.youtube.com/watch?v=pc3uQz06gO8&feature=youtu.be&app=desktop

The rationale for the development of MR-Base was to provide easy access to analysis-ready data and allow systematic application of Mendelian randomization methods. The tool was developed in the R statistical environment and has an application programming interface that controls user interaction with the underlying database, where curated GWAS data are stored and can be queried. Further technical details can be found in the existing MR-Base article ^3^. The aim of this article is to describe how the MR-Base platform can be used in practice (for example, for triangulation and transparency) and demonstrate these uses, through examples, to new audiences. It is complimentary to the existing MR-Base article, which focuses on describing and demonstrating MR-Base as a resource.

### Principles of Mendelian randomization

**Figure 1.**
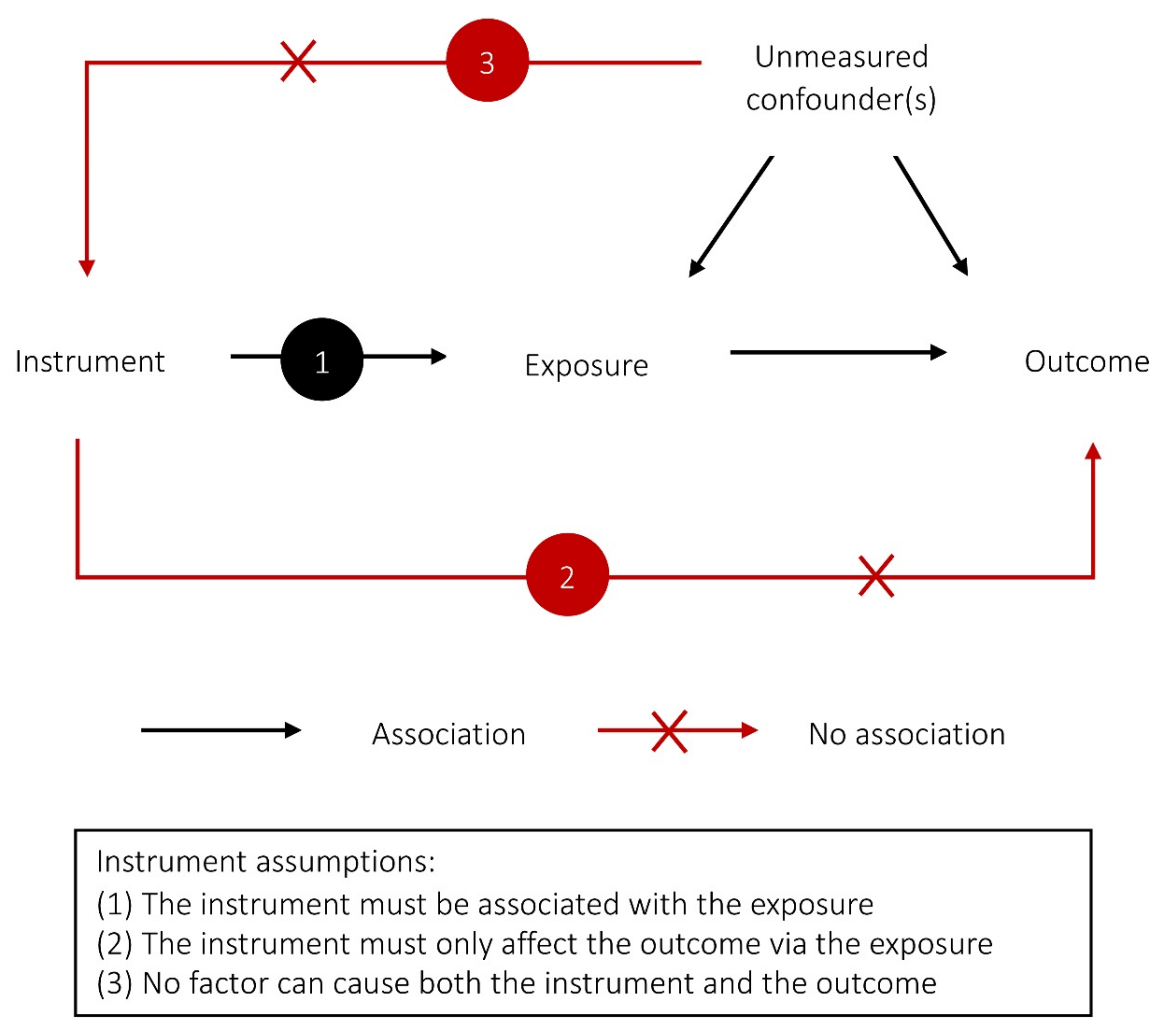
Overview of the instrument assumptions.

Mendelian randomization is a method to assess the causal effect of an exposure on an outcome using an instrument, defined by one or more SNPs, as a proxy for the exposure. The SNPs are used as instrumental variables and must meet three conditions: (i) they must be associated with the exposure; (ii) they must only affect the outcome via the exposure; and (iii) there must be no factor that causes both the SNP and outcome. These conditions are known as the instrumental variable assumptions and are illustrated in Figure 1. SNPs are plausible instruments because they are determined at conception and generally cannot be subsequently affected by the environment ^4^. If these assumptions hold, then Mendelian randomization effect estimates are unlikely to be due to confounding or reverse causation. However, Mendelian randomization is still subject to important limitations (see Limitations of Mendelian randomization).

Methodological advances mean that Mendelian randomization can be implemented using summary statistics from GWAS, without individual level data ^5^. This requires SNP-exposure associations and SNP-outcome associations obtained from separate datasets and is known as two-sample Mendelian randomization ^5^. This is in contrast to one-sample Mendelian randomization, where both the exposure and outcome are measured in all individuals from the same sample. As a result, two-sample Mendelian randomization can exploit much larger sample sizes and estimate effects with higher precision than is typically possible using any single sample. It also allows access to large case control studies of disease outcomes that may not have measured the exposure of interest. This can be particularly beneficial when considering expensive or difficult to measure phenotypes, such as DNA methylation, metabolomics, proteomics, and gene expression ^6^. Note that the use of GWAS in this way for two-sample Mendelian randomization also requires us to make an additional assumption that the GWAS used are providing unbiased genetic estimates.

Detailed discussion of the theory and interpretation of Mendelian randomization results can be found elsewhere ^1, 6– 11^. Key definitions used throughout the present discussion are given in Table 1.

**Table 1.** Key definitions.

Term	Definition
Mendelian randomization	Mendelian randomization is a method to assess the causal effect of an exposure on an outcome using an instrument, defined by one or more single nucleotide polymorphisms, as a proxy for exposure.
Genome-wide association study (GWAS)	Genome-wide association studies identify the genetic variants that are associated with a given phenotype.
Single nucleotide polymorphism	A single nucleotide polymorphism is a difference in the DNA nucleotides between individuals.
Triangulation	“The practice of obtaining more reliable answers to research questions through integrating results from several different approaches, where each approach has different key sources of potential bias that are unrelated to each other ^13^.”
Pleiotropy	Pleiotropy is when genetic variants effect multiple phenotypes that appear to be unrelated.
Horizontal pleiotropy	Horizontal pleiotropy occurs when the outcome is affected by the instrument single nucleotide polymorphism(s) through a pathway that is independent of the exposure and invalidates the second Mendelian randomization assumption. This is opposed to vertical pleiotropy, which occurs when the instrument single nucleotide polymorphism(s) are associated with other phenotypes that occur between exposure and outcome or after the outcome of interest and does not invalidate the Mendelian randomization assumptions.
Collider bias	A form of bias introduced as a result of conditioning on a variable that is both a consequence of the exposure and the outcome and hence changing the relationship between them.
Genome-wide significance	A conventional threshold, defined as p-values less than 5e-8, that is commonly used to determine which genetic variants are ‘hits’ in a genome-wide association study.
Allele harmonization	Allele harmonization is the process of specifying the effect and other alleles in the same way in both the outcome and exposure data.
Clumping	Clumping is a method for identifying the independent signals among correlated SNPs.
Linkage disequilibrium	Two genetic variants are in linkage disequilibrium if their alleles are associated.
Heterogeneity	Heterogeneity is defined as the variation in the causal estimate across SNPs.
Palindromic single nucleotide polymorphism (SNP)	A SNP is described as palindromic if the pair of alleles on the forward-strand are the same as the pair of alleles on the reverse strand (i.e. G/C or A/T SNPs).
Minor allele frequency (MAF)	The MAF is a measure of how common the least common allele is for a given genetic variant.
Funnel plot	Funnel plots present the effect estimates against a measure of precision – in the case of MR-Base, the inverse standard error of the instrument – to allow visual assessment of heterogeneity.
No measurement error (NOME) assumption	The NOME assumption assumes that the variance of the instrument-exposure association is negligible and so can be ignored.
Quantitative trait locus (QTL)	A QTL is a DNA variant associated with the variation that is observed in a phenotype.
Zero modal pleiotropy assumption (ZEMPA)	An assumption that the mode of the bias terms for individual instruments is zero.
Instrument strength independent of direct effect assumption (INSIDE)	An assumption that there is zero correlation between the SNP-exposure associations (i.e. the instrument strength) and the SNP-outcome associations (i.e. the direct effect of the instruments on the outcome) ^14^. Required for some MR methods, such as MR-Egger.

## Limitations of Mendelian randomization

Mendelian randomization is subject to several limitations. For example, effect sizes may not be indicative of the effects of a clinical intervention later in life. This can occur for several reasons, such as cumulative exposure, where Mendelian randomization estimates may reflect the effect of lifelong exposure, or time-dependent exposure, where intervention outside of a critical period does not have an effect despite the Mendelian randomization estimates suggesting an effect exists. Mendelian randomization estimates may also be affected by issues such as horizontal pleiotropy, whereby the SNPs chosen to proxy the exposure may affect the outcome by pathways other than the exposure of interest leading to biased results ^1, 6– 12^. Furthermore, investigators are reliant on relevant data being made available and appropriate methods being developed. For instance, it is currently difficult to study prognostic factors due to the lack of GWAS conducted for disease progression outcomes and the susceptibility of current methods to collider bias ^15^. Selection bias, which occurs when the individuals included within the analysis are not representative of the population that you are trying to study, can also be an issue ^16^. Careful consideration of the GWAS used for two-sample Mendelian randomization is therefore encouraged to minimize the effect of this bias on estimates. An additional limitation, specific to two-sample Mendelian randomization, is the requirement for no sample overlap. While using different samples for the exposure and outcome data is an advantage of this method as it can increase power, it can be difficult to determine whether individual participants appear in both datasets. If they do, simulations have suggested there is a potential for “substantial bias and inflated Type 1 error rates ^17^”. While MR-Base includes multiple sensitivity analyses that aim to address some of these limitations, such as horizontal pleiotropy. Others, such as sample overlap, are not accounted for and therefore must be considered carefully by users of this tool. Further details and limitations of Mendelian randomization can be found in the literature ^1, 6– 11^.

### Potential applications of MR-Base

MR-Base ^2^ is suitable for a broad range of applications and, consequently, is intended for use by a broad range of professionals. These include clinical and non-clinical researchers, public health specialists, policy makers and those in the pharmaceutical industry. Some of the key ways in which the platform can be used are summarised below and specific use cases are discussed in the *Use cases* section.

- MR-Base can be used to rapidly implement two-sample Mendelian randomization to investigate potential risk and prognostic factors (use cases 1 and 2) and evaluate potential drug targets (use case 3). The GWAS database and online analytical platform provided by MR-Base allow two-sample Mendelian randomization to be implemented quickly and easily to test associations for a range of traits (behavioural, physiological, hormonal, epigenomic, metabolomic, microbiomic) in relation to outcomes. This can be used without the need to generate new data, for example, when exploring new research ideas. Note that these investigations are subject to the relevant data being made available and appropriate methods being developed (see Limitations of Mendelian randomization). However, new data are regularly added to the database and the platform is regularly updated to incorporate the latest methods, which should help to overcome these issues in the future.
- MR-Base can conduct sensitivity analyses (use case 2). As with all analytical methods, Mendelian randomization methods are based on assumptions which may not hold. MR-Base offers many Mendelian randomization methods for investigators to choose from and conducts several standard sensitivity analyses that allow relaxation of the assumptions and provide ways of assessing potential pleiotropy. Some of the more commonly used Mendelian randomization methods are selected by default in the platform, including the Wald ratio ^18^, MR-Egger ^14^ and the inverse variance weighted method ^14^. Use of multiple methods is recommended as they differ in their strengths, limitations and efficiency. For example, MR-Egger has been developed to detect assumption violations such as invalid instruments due to pleiotropy but can lack precision. The best way to assess the reliability of the causal estimates obtained from Mendelian randomization is to triangulate across multiple Mendelian randomization methods and with findings from non-Mendelian randomization study designs ^13^. This is demonstrated in use case 2. Summary tables for the methods and the output of Mendelian randomization analyses conducted using MR-Base (as of October 2018) are provided in Table 2 and Table 3, respectively. The platform continues to be under active development and, as highlighted before, new methods are added as they arise.
- MR-Base can replicate results (use cases 2 and 3). MR-Base can be used to replicate the results of studies, regardless of whether they originally used Mendelian randomization or MR-Base, if relevant GWAS are available. This may be useful in several situations, including when appraising studies in the literature.
- MR-Base can be used to support triangulation of evidence (use case 2). Triangulation has been defined as “the practice of obtaining more reliable answers to research questions through integrating results from several different approaches, where each approach has different key sources of potential bias that are unrelated to each other ^13^.” Mendelian randomization, implemented in MR-Base, can be linked with other designs which are intended to reveal biases (for example, a negative control study) or exploit different confounder structures (for example, a cross-context comparison as a source of evidence in a triangulation framework).
- MR-Base can enable transparency (use case 1). MR-Base has been developed to encourage transparency by providing the analysis code needed to replicate the analysis in the output. Further to this, studies that use data from the platform can be directly replicated by others as they can access the same data that has been formatted in a consistent manner via the provided allele harmonization procedures.

**Table 2.** Overview of MR methods available in MR-Base.

Method	Details	References
Wald ratio	The Wald ratio method is also known as the ratio of coefficients method. It divides the regression coefficient of the instrument on the outcome by the regression coefficient of the instrument on the exposure and can be used when only one instrument SNP is available.	18
Maximum likelihood	This method maximizes the likelihood of a model, which is based on the exposure- outcome relationship and the distribution of the estimates of the genetic association, to obtain a causal estimate.	19
MR Egger regression	MR Egger calculates Wald ratios for each of the instruments and combines the results using an adapted Egger regression. The causal effect is the Egger regression slope coefficient and the intercept is an estimate of the average pleiotropic effect across instruments. Bootstrapping can help to improve the reliability of standard error estimates for non-zero causal effects.	14
MR Egger (bootstrap)		
Simple median	These methods calculate Wald ratios for each of the instruments and select the median value (according to the specified method) as the causal estimate. They provide valid estimates when more than half of the SNPs satisfy the instrumental variable assumptions.	20, 21
Weighted median		
Penalised weighted median		
Inverse variance weighted	This method calculates the Wald ratio for each of the instruments and combines the results using an inverse-variance weighted meta-analysis approach. The slope from this approach can be interpreted at the causal effect of the exposure on the outcome. The variance of the effect can be estimated using either a fixed or multiplicative random effects model. The latter is usually implemented unless there is under-dispersion in the effect estimates, in which case a fixed effects model is used.	19, 22
Inverse variance weighted (multiplicative random effects)		
Inverse variance weighted (fixed effects)		
Simple mode	The mode-based methods use the causal effect estimates for individual SNPs to form clusters. The causal effect estimate is then taken as the causal effect estimate from the largest cluster of SNPs. The weighted mode methods use the same process but assign weights to each SNP. Mode-based methods require ZEMPA, which states that the mode of the bias terms for the individual instruments is zero.	23
Weighted mode		
Weighted mode (NOME)		
Simple mode (NOME)		

**Table 3.** Overview of the tables and graphs included in the MR-Base platform.

Tab	Details
MR results	A table with the causal estimates resulting from each MR method that was implemented. See Table 4 for an example based on use case 2. Estimates are presented in the units of the exposure SNP(s). Estimates are beta coefficients for the outcome and should be exponentiated if the unit of the outcome was a log odds ratio. P-values are calculated using a t-distribution.
Heterogeneity statistics	A table with statistics indicating the variation in the causal estimate across SNPs, i.e. heterogeneity. Lower heterogeneity indicates better reliability of results.
Causal direction test	The results of a test that uses variation explained in both the exposure and outcome to assess whether the direction of the results is likely to be correct. Note the test cannot determine whether a causal association exists.
Horizontal pleiotropy	The Egger regression intercept with its standard error and a p-value.
Single SNP analysis	A summary graph showing the individual effects of SNPs, calculated using the Wald ratio, along with the overall results to assess the consistency across SNPs. See Figure 2 for an example.
Method comparison plot	A graphical representation of the results given under the ‘MR results’ tab. This graph shows the effect of the SNP(s) on exposure against the effect of the SNP(s) on the outcome. The graph is structured so that the effect of the SNP(s) on the exposure is always positive and the effect of the SNP(s) on the outcome is directed accordingly. See Figure 3 for an example based on use case 2.
Leave-one-out analysis	A graph showing the results of MR analyses using the inverse variance weighted method when leaving one SNP out each time. This analysis can be used to assess whether the SNPs are consistent in terms of their effect on the overall outcome or whether the results are being driven by a single outlying SNP. See Figure 4 for an example based on use case 2.
Funnel plot	A graph to visually assess heterogeneity, particularly horizontal pleiotropy. Horizontal pleiotropy is likely if points are spread. Directional horizontal pleiotropy may be present if the graph is not symmetrical. See Figure 5 for an example based on use case 2.

## Methods

### Implementation

MR-Base ^2^ can perform two-sample Mendelian randomization and provide summary statistics from a range of GWAS for this purpose. As highlighted previously, it can be accessed through a web application or as an R package. Data can either be accessed through the platform or be uploaded by the user, both of which are demonstrated in the following *Use cases* section. Data harmonization between the SNP-exposure associations and the SNP-outcome associations and Mendelian randomization are then performed according to options specified by the user using buttons on the web application or commands for the R package.

### Operation

The web application can be accessed from any platform that allows the use of a java-script compatible graphical web browser. The R package can be accessed from any platform where R version 3.5 or later can be installed. Step-by-step instructions for the web application and code for the R package are available for each of the use cases (see *Software availability*) ^24^. A generalized workflow for using the MR-Base web interface is provided in Box 3.

Box 3. **Generalized workflow for using the MR-Base web interface**

Mendelian randomization analyses can be performed using the MR-Base web interface as detailed below:

1. Access the platform ( http://www.mrbase.org/) and sign the data access agreement using a Google account.
2. Define the exposure according to one of the following options:
  a. By selecting the relevant GWAS from an existing source, such as the MR-Base GWAS catalog. This is demonstrated in use cases 1 and 2.
  b. By uploading an instrument file, specifying the delimiter for the file and filling in the form to map the column names to those supplied in the file. Columns not included in the file can be left blank in the mapping. This is demonstrated in use case 3.
3. Define the outcome by selecting the relevant GWAS from the MR-Base GWAS catalog.
4. Specify the analysis settings:
  • Set linkage disequilibrium (LD) clumping preference – by default this will be ‘Do not check for LD between SNPs’.
  • Set linkage disequilibrium proxies preference – by default this will be to use proxies with a minimum linkage disequilibrium R squared value of 0.8 and allow palindromic SNPS with a minor allele frequency threshold up to 0.3.
  • Set allele harmonisation preference – by default this will be ‘Attempt to align strands for palindromic SNPs’. If used, this setting will remove palindromic SNPs with minor allele frequencies close to 0.5 as the effect allele will be ambiguous.
  • Select the methods for analysis – by default this will be the Wald ratio, MR Egger, weighted median, inverse variance weighted and weighted mode methods.
5. Select the ‘perform MR analysis’ button and save the results, including the citations that are to be referenced in any published work arising from this analysis, on the following screen.

Note that the MR-Base web interface will provide the analysis code as an output if you wish to recreate your analysis in R. Also, note that there will be no graphical results produced for single SNP instruments as the sensitivity analyses, which are illustrated in the graphs, can only be conducted when there are multiple SNPs.

## Use cases

We discuss three studies that demonstrate important applications of Mendelian randomization and highlight the benefits of using MR-Base for these types of analyses in the following sections.

### Use case 1: subjective wellbeing and cardiometabolic health

Our first use case demonstrates the rapid implementation of Mendelian randomization using MR-Base to investigate a risk factor proposed in the observational literature and enable transparency of the Mendelian randomization study. The specific workflow for this case study is provided, alongside the necessary code, data and results, on GitHub (see *Software availability*) ^24^. It is based on work by Wootton *et al.* that used MR-Base to investigate the association between subjective wellbeing and 11 measures of cardiometabolic health ^25^. It has been reproduced based on information in the paper and, in particular, their code on GitHub. Studies with data on both subjective wellbeing and measures of cardiometabolic health are rare. Therefore, the authors chose to use two-sample Mendelian randomization so that they could use separate samples for their exposure (sample 1) and outcome (sample 2) phenotypes and use UK Biobank as a single-sample Mendelian randomization sensitivity analysis. In addition to this, the largest available GWAS of subjective wellbeing at the time of the study had identified just three SNPs to instrument this phenotype at the conventional genome-wide significance level and only one of these three SNPs replicated in an independent sample. Consequently, the authors used a lower p-value threshold of P < 5×10 ^-5^ to increase the number of SNPs in their instrument. This potentially increases their instrument strength but may also increase their susceptibility to weak instrument bias or pleiotropy.

It was straightforward to use two-sample Mendelian randomization with MR-Base in this study as it allowed the authors to consider multiple outcomes simultaneously and to use summary data that was already formatted for analysis. It also allowed specification of their preferred p-value threshold for the instrument. To make allowance for the non-independence of the selected SNPs, it was necessary to prune the SNPs to determine an independent set for the analysis. MR-Base allows users to select independent SNPs through a process known as ‘clumping’, which identifies independent signals by considering the linkage disequilibrium between SNPs. Linkage disequilibrium refers to the allelic association between groups of SNPs, which are typically located in a similar region of the genome. Failure to consider the linkage disequilibrium between SNPs can lead to overestimation of instrument strength and overly precise effect estimates. MR-Base overcomes this by picking the SNP from the group of SNPs in linkage disequilibrium that has the strongest evidence of association with the exposure for use in the Mendelian randomization analysis. In the Wootton *et al.* study, clumping reduces the subjective wellbeing instrument from 724 SNPs to 84 SNPs, highlighting the importance of this step in the analysis.

### Use case 2: systolic blood pressure and coronary heart disease

**Figure 2.**
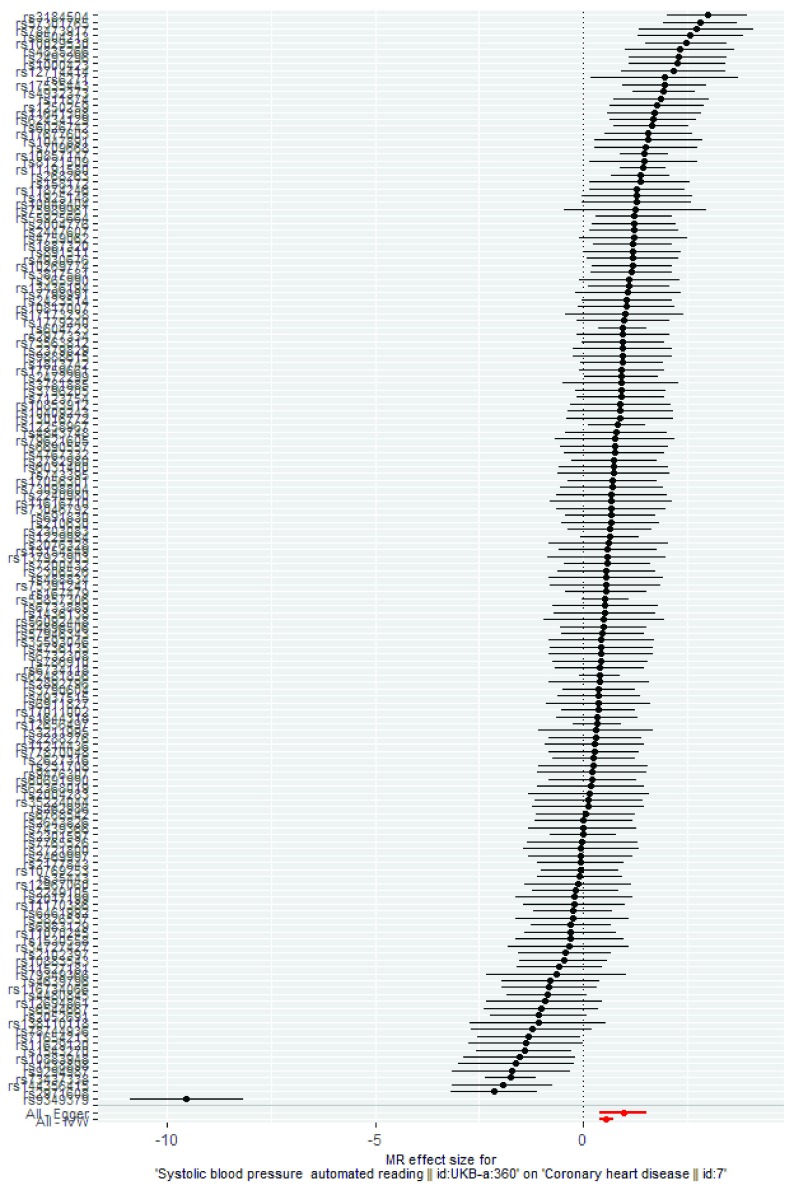
Exemplar single SNP analysis plot based on use case 2.

The second use case demonstrates how MR-Base can be used to conduct sensitivity analyses and triangulate evidence. The specific workflow for this case study is provided, alongside the necessary code, data and results, on GitHub (see *Software availability*) ^24^. Sample code for using the ‘TwoSampleMR’ R package based on this use case is provided in Box 4 and exemplar output is provided in Table 4 and Figure 2– Figure 5. It is based on work by Ference *et al.* that examined the effect of systolic blood pressure on coronary heart disease ^26^. Here, we recreate the Mendelian randomization component of the work. This can be triangulated with evidence from meta-analyses of prospective observational studies and randomized controlled trials. These meta-analyses found that lower systolic blood pressure reduced risk of coronary heart disease with odds ratios of 0.75 (95% CI: 0.71 to 0.78; p = 0.006) and 0.83 (95% CI: 0.76 to 0.90; p = 0.001), respectively. A full discussion of the triangulation element of this research is provided by Lawlor *et al.* ^13^

Box 4. **Sample code for using the ‘TwoSampleMR’ R package based on use case 2**

# Load the TwoSampleMR package

library(TwoSampleMR)

# List the outcomes available in MR-Base

ao <- available_outcomes()

# Extract the instruments from the systolic blood pressure GWAS (ID: 'UKB-a:360')

exposure_dat <- extract_instruments(c('UKB-a:360'))

# Extract the outcome data from the coronary heart disease GWAS (ID: 7)

outcome_dat <- extract_outcome_data(exposure_dat$SNP, c('7'),
                                    proxies = 1, rsq = 0.8, align_alleles = 1,
                                    palindromes = 1, maf_threshold = 0.3)

# Harmonize the exposure and outcome data

dat <- harmonise_data(exposure_dat, outcome_dat, action = 2)

# Perform MR analysis

mr_results <- mr(dat)

**Table 4.** Exemplar MR results table based on use case 2.

id.exposure	id.outcome	outcome	exposure	method	nsnp	b	se	pval
UKB-a:360	7	Coronary heart disease || id:7	Systolic blood pressure automated reading || id: UKB-a:360	MR Egger	157	0.9711	0.2917	0.001091
UKB-a:360	7	Coronary heart disease || id:7	Systolic blood pressure automated reading || id: UKB-a:360	Weighted median	157	0.571	0.07664	9.226e-14
UKB-a:360	7	Coronary heart disease || id:7	Systolic blood pressure automated reading || id: UKB-a:360	Inverse variance weighted	157	0.5663	0.0905	3.924e-10
UKB-a:360	7	Coronary heart disease || id:7	Systolic blood pressure automated reading || id: UKB-a:360	Weighted mode	157	0.571	0.1744	0.00131

**Figure 3.**
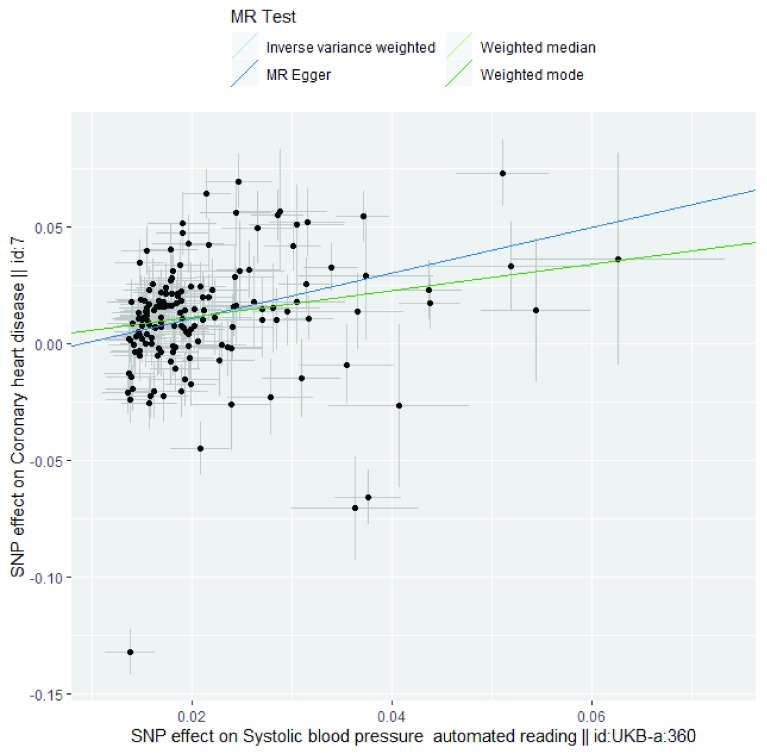
Exemplar method comparison plot based on use case 2.

**Figure 4.**
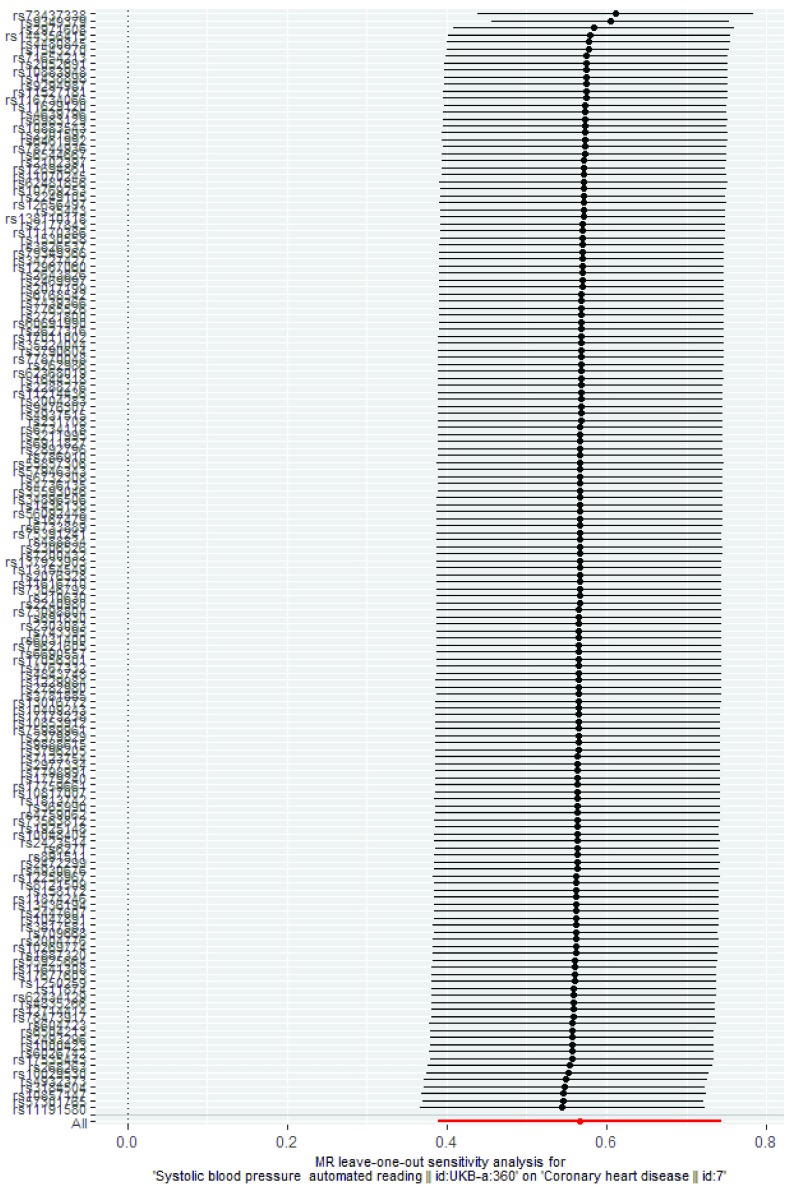
Exemplar leave-one-out analysis plot based on use case 2.

**Figure 5.**
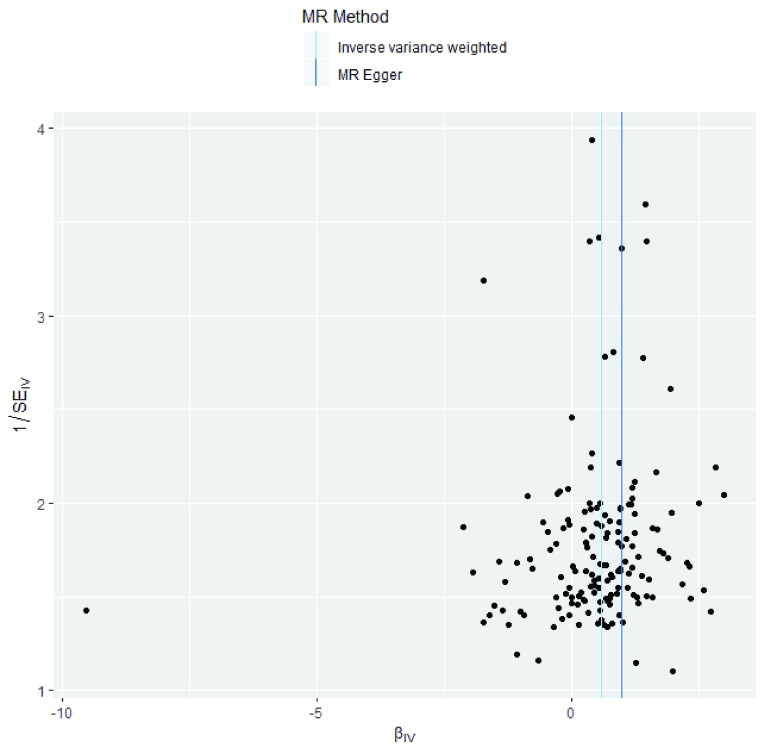
Exemplar funnel plot based on use case 2.

In our results, as with the original paper, we were concerned about directional pleiotropy, which occurs when genetic variants affect the outcome independently of the exposure. This is because large GWAS, such as the GWAS of systolic blood pressure we use here, may identify SNPs of unknown function ^27^. To assess the effect of this upon our results, we can look at the MR-Egger regression intercept provided by default on the MR-Base web application and calculable using the TwoSampleMR package for R. The intercept provides an estimate of the magnitude of horizontal pleiotropy and in our case is -0.0087 (SE: 0.0059; p = 0.147). This suggests limited evidence for directional pleiotropy among our results. We also used several Mendelian randomization methods for our analysis as a further sensitivity analysis and found consistent results for the effect of increased systolic blood pressure on coronary heart disease, regardless of the method used (IVW - OR: 1.76, 95% CI: 1.48 to 2.10, p = 3.92e-10; MR Egger - OR: 2.64, 95% CI: 1.49 to 4.68, p = 1.09e-03; Weighted median – OR: 1.77, 95% CI: 1.52 to 2.05, p = 3.92e-10; Weighted mode – OR: 1.77, 95% CI: 1.26 to 2.49, p = 1.31e-3). Note that the estimates returned by MR-Base are beta coefficients for the outcome. Binary outcomes are commonly reported as log odds ratios and so will need to be exponeniated in order to obtain an odds ratio, as was done here.

### Use case 3: HMGCR and type 2 diabetes

Our final use case demonstrates how MR-Base can be used to replicate a study and appraise a potential pharmaceutical intervention. The specific workflow for this case study is provided, alongside the necessary code, data and results, on GitHub (see *Software availability*) ^24^. It is based on research by Swerdlow *et al.* that investigated the effect of 3-hydroxy-3-methylglutaryl-CoA reductase (HMGCR), the target of statins, on risk of type 2 diabetes ^28^. This study used a single SNP as an instrument: rs17238484. To demonstrate the features of MR-Base, we have uploaded the data necessary to define the instrument for this analysis, instead of using data already within the platform. These data were extracted from the 2013 GWAS by the Global Lipids Genetics Consortium ^29^. Once uploaded, the column names must be mapped to those used by MR-Base before the analysis can be run. If you are using the R package, there are equivalent commands that perform data formatting (see this guide). Although units are not required for the analysis to run, it is important that the units of the instrument-exposure and instrument-outcome effects are known, as this determines the interpretation of the effect estimate obtained by Mendelian randomization. 

If you use MR-Base, please cite the resource using reference ^3^. We also ask that you cite and acknowledge the studies that contributed the data and methodology used in your analysis.

## Conclusions

Mendelian randomization is a method for estimating causal effects of an exposure on an outcome that are unlikely to be due to confounding or reverse causation. The method has a broad range of applications, including the investigation of risk factors and the appraisal of potential targets for pharmaceutical intervention. MR-Base eases the implementation of this method by combining a database of GWAS results with an analysis interface to allow Mendelian randomization to be used in several ways, such as for transparency and replication. Consequently, novice users can now perform sophisticated, causal appraisals of exposures by implementing Mendelian randomization using this powerful but accessible tool.

## Data availability

### Underlying data

Source data for the use cases are available through the database integrated into the MR-Base platform. The database is large and contains data from over 20,000 GWAS; therefore, it is not possible to host this data on an external repository. The data can be accessed via the MR-Base web application or by using the TwoSampleMR package for R to interact with the application programming interface. Users are required to accept the data access agreement by logging in with a Google account before access to the data is granted.

## Software availability

MR-Base software:

- Software available from: http://www.mrbase.org/
- Source code available from: https://github.com/MRCIEU/TwoSampleMR
- Archived source code at time of publication: https://doi.org/10.5281/zenodo.3298001 ^2^
- License: GPL-3.0

Use cases workflow and code:

- Source code available from: https://github.com/MRCIEU/mrbase_casestudies
- Archived source code at time of publication: http://doi.org/10.5281/zenodo.3239316 ^24^
- License: MIT
