# Supplementary material for: Using the MR-Base platform to investigate risk factors and drug targets for thousands of phenotypes
Source: Wellcome Open Res. 2019 Nov 7;4:113. Originally published 2019 Jul 29. [Version 2] doi: 10.12688/wellcomeopenres.15334.2 (PMC6694718; doi:10.12688/wellcomeopenres.15334.2)
Supplement: Peer review history

# Peer review: Reviewer response for version 1

**DOI:** 10.21956/wellcomeopenres.16742.r36074

**Published:** 2019-09-23

**Authors:** Apostolos Gkatzionis

**Affiliations:** 1 MRC Biostatistics Unit, University of Cambridge, Cambridge, UK

The paper by Walker *et al*. provides an introduction to the already popular MR-Base platform, a software tool developed by the authors for accessing GWAS results and conducting Mendelian randomization analyses in a fast and efficient way. Prior to reading the paper, I have not had the opportunity to use MR-Base. While reviewing, I used the web application to reproduce the authors’ Use Cases 1 and 2 as a tutorial. In my experience, the software is user-friendly, straightforward in its implementation and produces a concise and informative report. Likewise, the paper serves well as an instructions manual that is easy for applied practitioners to follow. It is therefore a welcome addition to the literature.

 My comments are minor and mainly concerned with the presentation of the paper:

- I agree with previous reviewers that the article would benefit from a short discussion of the challenges involved in performing Mendelian randomization. The MR-Base software efficiently automates the process of implementing an MR analysis, which is naturally very useful for practical purposes. However, this makes it easy to ignore some of the assumptions that underpin Mendelian randomization and limitations of the approach. Especially topics that are not automatically addressed by MR-Base, such as selection bias and the need for SNP-exposure and SNP-outcome associations to be obtained from similar populations, could be highlighted to alert applied practitioners using the software to potential limitations of their analyses.
- As suggested by the authors, MR-Base can greatly contribute to promoting transparency and reproducibility of research results in Mendelian randomization. Given that the software is already quite popular, it could be useful if the authors incorporated a searchable database (similar to the GWAS catalogue) of published Mendelian randomization analyses that were conducted using MR-Base. This would, for example, allow researchers who want to investigate a specific trait to easily access previous research findings concerning causal relationships between that trait and other common risk factors or outcomes.
- It is helpful that the paper is complemented with links to GitHub pages, containing instructions on how to implement the authors’ analysis for each of the three Use Cases. Nevertheless, I think it could improve the presentation of the manuscript if the authors incorporated some of the results tables and associated graphs obtained from MR-Base into the paper for illustration purposes.

Overall, the MR-Base software and associated GWAS catalogue are valuable tools for applied researchers and can greatly simplify the process of conducting Mendelian randomization. I enjoyed learning about them for this review and look forward to using them in my research.

I confirm that I have read this submission and believe that I have an appropriate level of expertise to confirm that it is of an acceptable scientific standard.

# Response

**Published:** 2019-11-04

**Authors:** Venexia Walker

**Affiliations:** University of Bristol, UK

Thank you for your comments on our article. We have responded to each of them below.

*“I agree with previous reviewers that the article would benefit from a short discussion of the challenges involved in performing Mendelian randomization. The MR-Base software efficiently automates the process of implementing an MR analysis, which is naturally very useful for practical purposes. However, this makes it easy to ignore some of the assumptions that underpin Mendelian randomization and limitations of the approach. Especially topics that are not automatically addressed by MR-Base, such as selection bias and the need for SNP-exposure and SNP-outcome associations to be obtained from similar populations, could be highlighted to alert applied practitioners using the software to potential limitations of their analyses.”*

**

 We have added a section titled ‘Limitations of Mendelian randomization’ to the article, which includes discussion of selection bias and the requirement for non-overlapping samples in two-sample Mendelian randomization. It reads: “Mendelian randomization is subject to several limitations. For example, effect sizes may not be indicative of the effects of a clinical intervention later in life. This can occur for several reasons, such as cumulative exposure, where Mendelian randomization estimates may reflect the effect of lifelong exposure, or time-dependent exposure, where intervention outside of a critical period does not have an effect despite the Mendelian randomization estimates suggesting an effect exists. Mendelian randomization estimates may also be affected by issues such as horizontal pleiotropy, whereby the SNPs chosen to proxy the exposure may affect the outcome by pathways other than the exposure of interest leading to biased results ^1, 5–11^.  Furthermore, investigators are reliant on relevant data being made available and appropriate methods being developed. For instance, it is currently difficult to study prognostic factors due to the lack of GWAS conducted for disease progression outcomes and the susceptibility of current methods to collider bias ^13^. A related issue is selection bias, which often occurs due to the presence of a collider. This form of bias is present when the individuals included within the analysis are not representative of the population that you are trying to study. [ https://doi.org/10.1093/ije/dyy202]  Careful consideration of the GWAS used for two-sample Mendelian randomization is therefore encouraged to minimize the effect of this bias on estimates. An additional limitation, specific to two-sample Mendelian randomization, is the requirement for no sample overlap. While using different samples for the exposure and outcome data is an advantage of this method as it can increase power, it can be difficult to determine whether individual participants appear in both datasets. If they do, simulations have suggested there is a potential for “substantial bias and inflated Type 1 error rates”. [ https://doi.org/10.1002/gepi.21998]  While MR-Base includes multiple sensitivity analyses that aim to address some of these limitations, such as horizontal pleiotropy. Others, such as sample overlap, are not accounted for and therefore must be considered carefully by users of this tool. Further details and limitations of Mendelian randomization can be found in the literature ^1, 5–10^.”

*“As suggested by the authors, MR-Base can greatly contribute to promoting transparency and reproducibility of research results in Mendelian randomization. Given that the software is already quite popular, it could be useful if the authors incorporated a searchable database (similar to the GWAS catalogue) of published Mendelian randomization analyses that were conducted using MR-Base. This would, for example, allow researchers who want to investigate a specific trait to easily access previous research findings concerning causal relationships between that trait and other common risk factors or outcomes.”*

**

 We do not require users to directly notify us of published work that has used MR-Base however, we do ask that they cite the original MR-Base article in any published research. Reported research findings can therefore be found by looking through the citations for this article. There are currently no plans to include a searchable database of published Mendelian randomization analyses because of the large amount of manual curation of studies that would be needed to create such a database.

*“It is helpful that the paper is complemented with links to GitHub pages, containing instructions on how to implement the authors’ analysis for each of the three Use Cases. Nevertheless, I think it could improve the presentation of the manuscript if the authors incorporated some of the results tables and associated graphs obtained from MR-Base into the paper for illustration purposes.”*

**

 We agree that exemplar tables and graphs that demonstrate the output from MR-Base would improve the article. We have therefore added exemplar output for use case 2 – see Table 4 and Figures 2-5.

# Peer review: Reviewer response for version 1

**DOI:** 10.21956/wellcomeopenres.16742.r36078

**Published:** 2019-08-14

**Authors:** C. Mary Schooling

**Affiliations:** 1 Graduate School of Public Health and Health Policy, City University of New York, New York, NY, USA; 2 School of Public Health, The University of Hong Kong, Hong Kong SAR, China

This is an excellent instructional resource concerning use of Mendelian randomization (MR) and MR-Base. In that context it would be very helpful if the paper included material explaining the interpretation of MR-Base estimates in relation to causal inference, consolidated important limitations of MR-Base into one comprehensive section and reflected more on the interpretation of the MR estimate. Some of this information on limitations and interpretation of MR is already in the paper, but could be consolidated and made more comprehensive for the intended target audience, while the first item should be made clearer. Specifically,

1. The paper should make a clearer distinction between MR and causal inference. The paper explains that MR addresses confounding and reverse causation. However, valid causal inference requires estimates free from confounding and free from selection bias. So, the paper should draw the reader’s attention to this difference. As such, it would be better to explain throughout that MR gives unconfounded estimates rather than to equate MR with strengthening causal inference. As a general “health warning” it should also be pointed out that all MR studies depend on the assumption that the underlying genome wide association studies give unbiased genetic estimates. That is not to detract from the strengths of MR-Base and MR but just to be clear for the reader.
2. The sections explaining the limitations of MR and MR-Base would be better consolidated under a heading “Limitations” for easy reference. The material in the second half of the paragraph on page 3 headed “Principles of Mendelian randomization” could be moved to this section. The sentences at the bottom of page 4 explaining that MR cannot be used to assess disease progression could be moved to this section. It might also be worth mentioning that MR may not give reliable estimates of the effects of risk factors in samples of patients. 
3. More information on the interpretation of the MR estimate would also be helpful. Many MR estimates rely on the INSIDE (instrument strength independent of the direct effect) assumption which seems to imply that the MR estimate means that the exposure of interest or a precursor sharing genetic predictors with the exposure could be causal.

MR-Base is a wonderful tool that generally gives the same results as MR conducted by hand or using other packages. In that context, there are a few clarifications that would be helpful.

1. MR-Base users may not realize that the results are given in beta coefficients for the outcome which needs to be exponentiated if the outcome is reported as logodds. Maybe this point could be added to Table 3.
2. MR-Base has sometimes given different p-values for MR-Egger than the MendelianRandomization package, possibly because MR-Base is using a t-distribution and MendelianRandomization is using a normal distribution to obtain these p-values. Maybe this this difference is no longer relevant but if it is still occurring it might be worth addressing it in some way.
3. The clump_data function of MR-Base could make it clear whether genetic variants are excluded because they are correlated with the ones retained or because they are not in the 1000 genomes catalog.

I confirm that I have read this submission and believe that I have an appropriate level of expertise to confirm that it is of an acceptable scientific standard.

# Response

**Published:** 2019-11-04

**Authors:** Venexia Walker

**Affiliations:** University of Bristol, UK

Thank you for your comments regarding our article. Please see our response below.

*“The paper should make a clearer distinction between MR and causal inference. The paper explains that MR addresses confounding and reverse causation. However, valid causal inference requires estimates free from confounding and free from selection bias. So, the paper should draw the reader’s attention to this difference. As such, it would be better to explain throughout that MR gives unconfounded estimates rather than to equate MR with strengthening causal inference.”*

**

 We have revised the language in the article to better distinguish MR and causal inference. For example: the first sentence, which read “Mendelian randomization (MR) is a method used to study the effects of risk factors and exposures on outcome traits using genetic variation” now reads “Mendelian randomization (MR) is a causal inference method used to study the effects of risk factors and exposures on outcome traits by exploiting genetic variation to address confounding and reverse causation”.

*“As a general “health warning” it should also be pointed out that all MR studies depend on the assumption that the underlying genome wide association studies give unbiased genetic estimates. That is not to detract from the strengths of MR-Base and MR but just to be clear for the reader.”*

 We have added this warning to the ‘Principles of Mendelian randomization’ section that introduces two-sample Mendelian randomization. It reads: “Note that the use of GWAS in this way for two-sample Mendelian randomization also requires us to make an additional assumption that the GWAS used are providing unbiased genetic estimates."

*“The sections explaining the limitations of MR and MR-Base would be better consolidated under a heading “Limitations” for easy reference. The material in the second half of the paragraph on page 3 headed “Principles of Mendelian randomization” could be moved to this section. The sentences at the bottom of page 4 explaining that MR cannot be used to assess disease progression could be moved to this section. It might also be worth mentioning that MR may not give reliable estimates of the effects of risk factors in samples of patients.”*

 We have added a section titled ‘Limitations of Mendelian randomization’ to the article that includes the sentences you highlighted from pages three and four. It reads: “Mendelian randomization is subject to several limitations. For example, effect sizes may not be indicative of the effects of a clinical intervention later in life. This can occur for several reasons, such as cumulative exposure, where Mendelian randomization estimates may reflect the effect of lifelong exposure, or time-dependent exposure, where intervention outside of a critical period does not have an effect despite the Mendelian randomization estimates suggesting an effect exists. Mendelian randomization estimates may also be affected by issues such as horizontal pleiotropy, whereby the SNPs chosen to proxy the exposure may affect the outcome by pathways other than the exposure of interest leading to biased results ^1, 5–11^.  Furthermore, investigators are reliant on relevant data being made available and appropriate methods being developed. For instance, it is currently difficult to study prognostic factors due to the lack of GWAS conducted for disease progression outcomes and the susceptibility of current methods to collider bias ^13^. A related issue is selection bias, which often occurs due to the presence of a collider. This form of bias is present when the individuals included within the analysis are not representative of the population that you are trying to study. [ https://doi.org/10.1093/ije/dyy202]  Careful consideration of the GWAS used for two-sample Mendelian randomization is therefore encouraged to minimize the effect of this bias on estimates. An additional limitation, specific to two-sample Mendelian randomization, is the requirement for no sample overlap. While using different samples for the exposure and outcome data is an advantage of this method as it can increase power, it can be difficult to determine whether individual participants appear in both datasets. If they do, simulations have suggested there is a potential for “substantial bias and inflated Type 1 error rates”. [ https://doi.org/10.1002/gepi.21998]  While MR-Base includes multiple sensitivity analyses that aim to address some of these limitations, such as horizontal pleiotropy. Others, such as sample overlap, are not accounted for and therefore must be considered carefully by users of this tool. Further details and limitations of Mendelian randomization can be found in the literature ^1, 5–10^.”

*“More information on the interpretation of the MR estimate would also be helpful. Many MR estimates rely on the INSIDE (instrument strength independent of the direct effect) assumption which seems to imply that the MR estimate means that the exposure of interest or a precursor sharing genetic predictors with the exposure could be causal.”*

 We deemed detailed discussion of the theory and interpretation of Mendelian randomization results to be beyond the scope of this article, which deals with the practical considerations when conducting this type of analysis using MR-Base. We refer readers at the end of the section ‘Principles of Mendelian randomization’ to the literature where further information on theory and interpretation of results can be found. However, we agree that the INSIDE assumption is an important consideration when using some Mendelian randomization methods and should have been included with the other assumptions in ‘Table 1. Key definitions.’ Consequently, we have added the following definition to that table: “An assumption that there is zero correlation between the SNP-exposure associations (i.e. the instrument strength) and the SNP-outcome associations (i.e. the direct effect of the instruments on the outcome). ^15^ Required for some MR methods, such as MR-Egger.”

*“MR-Base users may not realize that the results are given in beta coefficients for the outcome which needs to be exponentiated if the outcome is reported as logodds. Maybe this point could be added to Table 3.”*

**

 We have highlighted this point in Table 3 as suggested and reiterated it at the end of Use Case 2, which makes use of this transformation, with the following statement: “Note that the estimates returned by MR-Base are beta coefficients for the outcome. Binary outcomes are commonly reported as log odds ratios, as was the case here, and so will need to be exponeniated in order to obtain an odds ratio.”

*“MR-Base has sometimes given different p-values for MR-Egger than the MendelianRandomization package, possibly because MR-Base is using a t-distribution and MendelianRandomization is using a normal distribution to obtain these p-values. Maybe this difference is no longer relevant but if it is still occurring it might be worth addressing it in some way.”*

 As you mention, MR-Base uses a t-distribution to calculate p-values and other packages may use alternative distributions for this calculation. We have stated that this distribution is used in Table 3, so users are informed. Generally, we would encourage users to avoid using p-values to indicate the relevance of their findings and, instead, focus on effect estimates and confidence intervals.

*“The clump_data function of MR-Base could make it clear whether genetic variants are excluded because they are correlated with the ones retained or because they are not in the 1000 genomes catalog.”*

 As of 19 ^th^ September 2019, the ‘clump_data’ function states the reason for SNP removal. The TwoSampleMR package will need to be updated if the currently installed version was downloaded prior to this date.

# Peer review: Reviewer response for version 1

**DOI:** 10.21956/wellcomeopenres.16742.r36141

**Published:** 2019-08-08

**Authors:** Bernard M. Y. Cheung

**Affiliations:** 1 Department of Medicine, The University of Hong Kong, Hong Kong, China

**General comments:**

 The article by Walker *et al.* on using the MR-Base platform to investigate risk factors and drug targets for thousands of phenotypes is a welcome addition to the literature. Naturally, the authors wax lyrical about the MR-Base platform they created and developed. It is indeed an attractive platform in that it holds summary statistics on traits and health outcomes from over 20,000 GWAS. To search for relevant GWAS and retrieve the data from scratch would have taken a researcher weeks and months, whereas with this integrated platform, you can do this in a day once you have learnt how to use it. Another major advantage of this platform is that it is transparent and enables other researchers to confirm the results using the same database and the published analysis codes.

 The article is concise and focuses on the information the end-user would want. Notably, three case studies, or ‘use cases’, are discussed to illustrate the utility and advantages of MR-Base. The explanation of two-sample Mendelian randomization tends to be brief, but this is reasonable, as the authors have previously published extensively on the methodology.

 All in all, MR-Base is an exciting innovation that has already produced some important findings and the present article provides much practical information to enable interested researchers to explore and exploit this new research tool.

**Specific comments:**

- As ‘thousands of phenotypes’ is in the title, there should be some information on the quantity, breadth and depth of the phenotypic variables. Part of the information can be given as a table if appropriate.

- In lieu of Discussion, there is a paragraph of conclusions. Regardless of what the section is called, it would be nice if there were some discussions on the limitations of MR-Base.

- Would this database continue to be free or would it require subscription or payment in the future?

- In the Abstract, complementary was misspelt as complimentary.

I confirm that I have read this submission and believe that I have an appropriate level of expertise to confirm that it is of an acceptable scientific standard.

# Response

**Published:** 2019-11-04

**Authors:** Venexia Walker

**Affiliations:** University of Bristol, UK

Thank you for your feedback on our article. We have addressed your specific comments as follows.

*“As ‘thousands of phenotypes’ is in the title, there should be some information on the quantity, breadth and depth of the phenotypic variables. Part of the information can be given as a table if appropriate.”*

 We have intentionally provided the scale, but not a specific quantity, of phenotypes as the database continues to grow and there is no clear way to define distinct traits. However, we have added the following text to the introduction that highlights the breadth and depth of the phenotypic variables: “Traits include anthropometric measures, risk factors, metabolites, metals, vitamins, circulating proteins, disease outcomes, and disease intermediates (such as LDL cholesterol and systolic blood pressure), as well as DNA methylation and gene expression phenotypes.”

*“In lieu of Discussion, there is a paragraph of conclusions. Regardless of what the section is called, it would be nice if there were some discussions on the limitations of MR-Base.”*

**

 We have added a section titled ‘Limitations of Mendelian randomization’ to the article. It reads: “Mendelian randomization is subject to several limitations. For example, effect sizes may not be indicative of the effects of a clinical intervention later in life. This can occur for several reasons, such as cumulative exposure, where Mendelian randomization estimates may reflect the effect of lifelong exposure, or time-dependent exposure, where intervention outside of a critical period does not have an effect despite the Mendelian randomization estimates suggesting an effect exists. Mendelian randomization estimates may also be affected by issues such as horizontal pleiotropy, whereby the SNPs chosen to proxy the exposure may affect the outcome by pathways other than the exposure of interest leading to biased results ^1, 5–11^.  Furthermore, investigators are reliant on relevant data being made available and appropriate methods being developed. For instance, it is currently difficult to study prognostic factors due to the lack of GWAS conducted for disease progression outcomes and the susceptibility of current methods to collider bias ^13^. A related issue is selection bias, which often occurs due to the presence of a collider. This form of bias is present when the individuals included within the analysis are not representative of the population that you are trying to study. [ https://doi.org/10.1093/ije/dyy202]  Careful consideration of the GWAS used for two-sample Mendelian randomization is therefore encouraged to minimize the effect of this bias on estimates. An additional limitation, specific to two-sample Mendelian randomization, is the requirement for no sample overlap. While using different samples for the exposure and outcome data is an advantage of this method as it can increase power, it can be difficult to determine whether individual participants appear in both datasets. If they do, simulations have suggested there is a potential for “substantial bias and inflated Type 1 error rates”. [ https://doi.org/10.1002/gepi.21998]  While MR-Base includes multiple sensitivity analyses that aim to address some of these limitations, such as horizontal pleiotropy. Others, such as sample overlap, are not accounted for and therefore must be considered carefully by users of this tool. Further details and limitations of Mendelian randomization can be found in the literature ^1, 5–10^.”

*“Would this database continue to be free or would it require subscription or payment in the future?”*

 As stated in the disclaimers within the data access agreement, we do not guarantee that the Platform or any GWAS Data will always be available or interrupted. However, there are no current plans for the database to require a subscription or payment in the future.

*“In the Abstract, complementary was misspelt as complimentary.”*

 Thank you – we have corrected this misspelling.
